# Human Resistin Inhibits Myogenic Differentiation and Induces Insulin Resistance in Myocytes

**DOI:** 10.1155/2013/804632

**Published:** 2013-02-18

**Authors:** Chun Hua Sheng, Zhen Wu Du, Yang Song, Xiao Dong Wu, Yu Cheng Zhang, Mei Wu, Qian Wang, Gui Zhen Zhang

**Affiliations:** ^1^Department of Central Research, The Third Clinical College, Jilin University, Changchun, Jilin 130033, China; ^2^Cell Transplantation Center, The 208th Hospital of Chinese People's Liberation Army, Changchun, Jilin 130021, China

## Abstract

This study is aimed to investigate the effect of human resistin on myocyte differentiation and insulin resistance. The human resistin eukaryotic expression vector was stable transfected into C2C12 myocyte cells and was transiently transfected into COS7 cells. The effects of human resistin on cell proliferation, cell cycle, and myogenic differentiation of C2C12 cells were examined. Glucose uptake assays was performed on C2C12 myotubes by using [^3^H] 2-deoxy-D-glucose. The mRNA levels of insulin receptor (IR) and glucose transporter 4 (GLUT4) were evaluated by semiquantitative RT-PCR. Results showed by the C2C12 cells transfected with human resistin gene compared with that without transfecting gene are as follows: (1) cell proliferation was significantly promoted, (2) after inducing differentiation, the myotube's diameters and expression of desmin and myoglobin decreased, and (3) glucose uptake ratio was lowered and expression of IR and GLUT4 decreased. However, there was no significant difference in the glucose uptake ratio between C2C12 myotubes treated with a human resistin conditioned medium of COS7 cells and treated with control medium. These results suggest that maybe human resistin has not a direct role on insulin sensitivity of myocytes. However, maybe it impaired the insulin sensitivity of myocytes through suppressing myogenesis and stimulating proliferation of myoblasts.

## 1. Introduction

Resistin is known as an adipocyte-specific secretory factor (ADSF) that belongs to a gene family found in the inflammatory zone (FIZZ) or found in the resistin-like molecule (RELM) [1]. Mostly researches reach consensus on the effect of resistin inducing insulin resistance in rodents [1–6]. However, it is controversial in the studies evaluating resistin expression related to type 2 diabetes in humans [7–11]. It is necessary to investigate the effects of human resistin (hR) on the target cells of insulin action. The skeletal muscle is a major tissue to take up and utilize glucose in vivo. Although it is argued that whether resistin is expressed in a skeletal muscle or not [1, 10, 12, 13], it impacts glycometabolism by endocrine, autocrine, and paracrine ways so that it is conceivable that effects of resistin might contribute to the pathogenesis of impaired insulin sensitivity, it is significant to elucidate the effect of human resistin on myocytes. However, some reports considered the activity of recombinant human resistin may be lower than endogenous resistin [14–16], so we constructed a human resistin eukaryotic expression vector. C2C12 myoblast is originated from a skeletal muscle of mouse and forms myotube after inducing differentiation. COS7 cells, as the African green kidney cell line, express SV40 large T antigen and support the replication of plasmid vector which contains SV40 ori (including PcDNA3.1) or some mutant SV40 to obtain high-level expression of exogenous genes. As a high-performance transient expression of eukaryotic systems, it is a commonly used tool to study gene function. In this study, human resistin eukaryotic expression vector were stably transfected into C2C12 myoblast and transiently transfected into COS7 cells to examine the effect of hR on myocytes.

## 2. Materials and Methods

### 2.1. Plasmids' Construction

The pGEM-T human resistin (pGEM-T-hR) clone vector was kindly provided by professor Ying Sun (King's College London, London, UK). Human resistin (hR) gene was amplified by polymerase chain reaction (PCR) from the pGEM-T-hR clone vector. The primer sequences for hR amplification are as follow: forward primer: 5′-TCAGGTACCATGGCCATGAAAGCTCTCTGTCTCCTC-3′; reverse primer: 5′-TCGGAATTCTCAGGGCTGCACACGACAGC-3′. Both primers include additional leader sequences that corresponded to the KpnI and EcoRI restriction enzymes, respectively, that can direct hR gene cloning into the pcDNA3.1 plasmids (Pc-3.1) (Invitrogen). HR cDNA generated by PCR was confirmed by the gene sequencing and inserted into plasmid Pc-3.1 by gene recombination technology. The hR gene of the construct was named pcDNA-hR (Pc-hR). Pc-3.1 plasmid was used as a control vector. 

### 2.2. Cell Culture and Differentiation Assays

C2C12 and COS7 cells (American Type Culture Collection) were cultured in Dulbecco's minimal essential medium (DMEM) containing 25 mM glucose and 10% fetal bovine serum (FBS) (GIBCO), respectively. C2C12 cells were grown to 100% confluence with 10% FBS serum and then changed to 2% horse serum. By day 10, C2C12 cells were fused into myotubes.

### 2.3. Stable Transfection in C2C12 Cells

C2C12 cells were transfected with Pc-hR and Pc-3.1 plasmids by using Lipofectamine 2000 reagent (Invitrogen) following the protocol. 72 h after transfection, the medium was changed to the selection DMEM supplemented with 800 *μ*g/mL G418 (GIBCO). Three weeks later, the cell clones were screened and further cultured in DMEM medium containing 400 mg/mL G418.

### 2.4. Transient Transfection in COS7 Cells

The Pc-3.1 and Pc-hR plasmids were transfected, respectively, into COS7 cells by using Lipofectamine 2000 reagent. 72 h after transfection, the medium of COS7 cells was collected, centrifuged at 500 ×g for 5 min, and stored at 4°C as conditioned medium of hR for less than a week before use.

### 2.5. Immunocytochemistry and Immunofluorescence Staining

The immunocytochemical detection of hR expression was carried out in C2C12 cells as previously described [17] using mouse anti-human resistin monoclonal antibody (R&D Systems, Inc., 1 : 50 dilution). The cells were visualized using a microscope (Olympus) and the immunoreactivity was identified as brown cytoplasmic staining in cytoplasm. Immunofluorescence staining was performed to identify the expression of desmin and myoglobin in C2C12 myotubes using monoclonal mouse antibody to desmin (Sigma-Aldrich, 1 : 50 dilution) and myoglobin (R&D Systems, Inc., 1 : 50 dilution) and goat anti-mouse Cy3-conjugated secondary antibody (R&D Systems, Inc., 1 : 200 dilution). The cells were visualized under a fluorescence confocal microscopy (OLYMPUS confocal microscope FV500). The grayscale value of desmin and myoglobin expression and the myotubes diameters were measured from randomly selected microscope fields from five different wells of Pc-3.1-transfected and Pc-hR-transfected C2C12 myotubes, respectively, and analyzed by image analytical system HPIAS-1000. At least five fields were selected and the diameter of 150 myotubes was measured per well. 

### 2.6. Methyl Thiazolyl Tetrazolium (MTT) Assays to Determine Cell Growth

C2C12 cells transfected with Pc-3.1 and Pc-hR were seeded in 96-well tissue culture plates (2 × 10^3^ cells/well) respectively. At the indicated time points, the assays were performed as the manufacturer's instruction. 

### 2.7. Cell Cycle Analyses

C2C12 cells were cultured in culture flasks. After reaching 30% confluence, the cells were treated with DMEM without FBS for 24 h for synchronization. At the next day, cells were cultured with DMEM with 10% FBS. Forty-eight hours later, cells were trypsinized, fixed in 70% ethanol, and were analyzed by flow cytometry using a FACScan Flow Cytometer (Becton Dickinson, San Jose, CA, USA).

### 2.8. Glucose Uptake Assays

The measurements for glucose uptake were performed as described by Nakamori et al. [18]. Briefly, C2C12 myotubes (10 days after differentiation) transfected with pc-hR and pc-3.1 were grown in serum-free DMEM for 4 h and then incubated in the presence of insulin for 30 minutes at 37°C. Transport was started by adding 1 *μ*Ci [^3^H]2-Deoxy-D-glucose (Amersham Pharmacia Biotech) in 1 mL of the Krebs-Ringer phosphate buffer for 10 min at 37°C and stopped by rapidly washing for three times with ice-cold phosphate-buffered solution (PBS). Cells were lysed in 0.4 mL PBS containing 0.1% Triton X-100 for 45 min. Aliquots of the cell lysates were used for liquid scintillation counting and determination of protein content by the Bradford method. Nonspecific transport was assayed in the presence of 10 *μ*mol/L cytochalasin B (Sigma-Aldrich). In addition, C2C12 myotubes (10 days after differentiation) were incubated in conditioned medium of COS7 cells transfected with pc-3.1 and pc-hR, respectively, for 24 h. The cells were then changed to serum-free DMEM for 4 h and performed glucose uptake assays as above.

### 2.9. Semi Quantitative RT-PCR Analysis of mRNA Expression

Total RNA was extracted from cells with the use of TRIzol reagent (GIBCO). RNA (1 *μ*g) was reverse transcribed using RevertAid H Minus M-MuL V reverse transcriptase (Helena Biosciences, Europe, Sunderland, UK) and random hexamers in 20 *μ*L reaction volume, according to the manufacturer's instructions. The products (1 *μ*L of cDNA) were subjected to PCR with Ex Taq and primers. Primer sequences are as follow: IR: forward primer: 5′-ATGGACATCCGGAACAACCT-3′; reverse primer: 5′-TTGATGACAGTGGCAGGACA-3′ (the product was 493 bp), GLUT4: forward primer: 5′-CAACGTGGCTGGGTAGGCA-3′; reverse primer: 5′-ACACATCAGCCCAGCCGGT-3′ (the product was 587 bp), *β*-actin 1: forward primer: GATGGTGGGTATGGGTCAGAAGGA; reverse primer: GCTCATTGCCGATAGTGATGACCT (the product was 632 bp), and *β*-actin 2: forward primer: 5′-GGGACCTGACAGACTACCT-3′; reverse primer: 5′-CAGGATTCCATACCCAAG-3′ (the product was 268 bp). The PCR products were separated by electrophoresis on agarose gel, visualized by ethidium bromide staining, and quantitated with Gel Image Systems. The abundance of each specific mRNA was normalized on the basis of that of *β*-actin mRNA.

### 2.10. Western Blot Analysis

Cell supernatants of Cos7 cells transfected with Pc-3.1 and Pc-hR plasmids were collected and treated by western blot 72 h after transfection as described [19]. In brief, the proteins were subjected to SDS polyacrylamide gel electrophoresis, electroblotted onto Polyvinylidene fluoride (PVDF) membrane (Millipore), and immunodetected using mouse anti-human resistin monoclonal antibody and goat anti-mouse IgG horseradish peroxidase conjugate.

### 2.11. Statistical Analysis

Statistical analyses were carried out using SPSS (SPSS Inc. 12.0, Woking, UK) software. All qualitative data are representative of at least three independent experiments, with at least four wells per group per experiment. Quantitative data are presented as means ± SEM and were compared with Student's *t* test. *P* < 0.05 was considered statistically significant. 

## 3. Results

### 3.1. Construction of Recombinant Human Resistin (hR) Expression Vector and Transfection into Cell Lines

**Figure 1:**
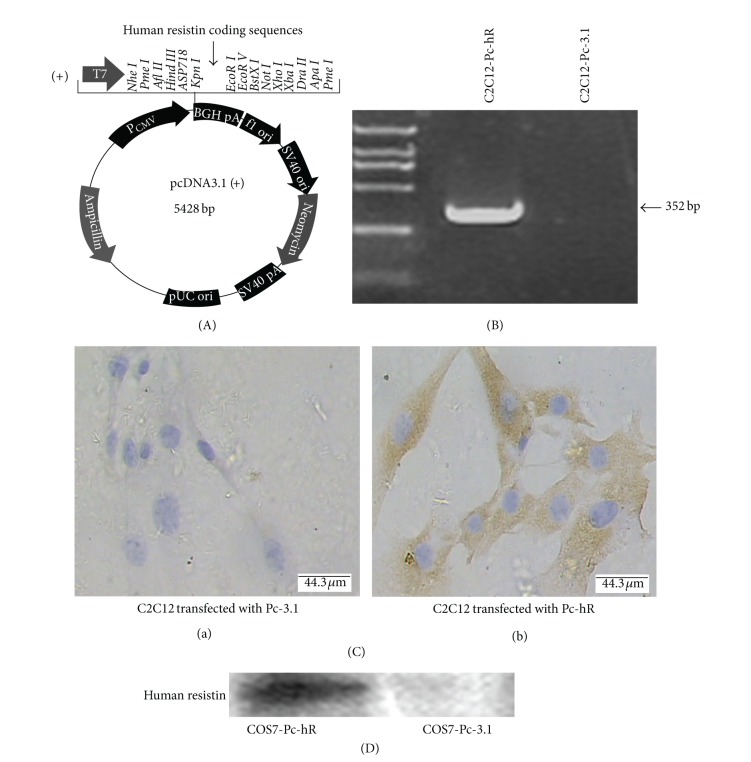
Construction of recombinant hR expression vector and transfection in cell lines. (A) A full length of hR coding sequence was inserted into PcDNA-3.1 (Pc-3.1). (B) The expression of hR mRNA was identified in C2C12 cells transfected with PcDNA-hR (Pc-hR). (C) Immunocytochemistry analysis showed hR immunoreactive protein in C2C12 cells transfected with Pc-hR. (D) Western blot analysis showed expression of hR in COS7 cells transfected with Pc-hR. C2C12-Pc-3.1: C2C12 cells transfected with Pc-3.1 plasmids; C2C12-Pc-hR: C2C12 cells transfected with Pc-hR plasmids.

The full length of cDNA encoding hR coding sequences (327 bp) was cloned into PcDNA3.1 (Pc-3.1) vectors (Figure 1(A)). The recombinant plasmid PcDNA-hR (Pc-hR) and the control plasmid Pc-3.1 were stably transfected into C2C12 cells and transiently transfected into COS7 cells. The expression of recombinant hR at mRNA and protein levels in C2C12 cells were identified by RT-PCR and immunocytochemistry respectively. Expression of hR mRNA and protein was observed in C2C12 cells transfected with Pc-hR plasmids (Figures 1(B) and 1(C-b)), but was not observed in C2C12 cells transfected with Pc-3.1 plasmids (Figures 1(B) and 1(C-a)). The expression of hR in COS7 cells was identified by Western blot (Figure 1(D)). HR expressed significantly in COS7 cells transfected with Pc-hR whereas not expressed in control cells.

### 3.2. Effects of hR on Proliferation and Cell Cycle of C2C12 Cells

**Figure 2:**
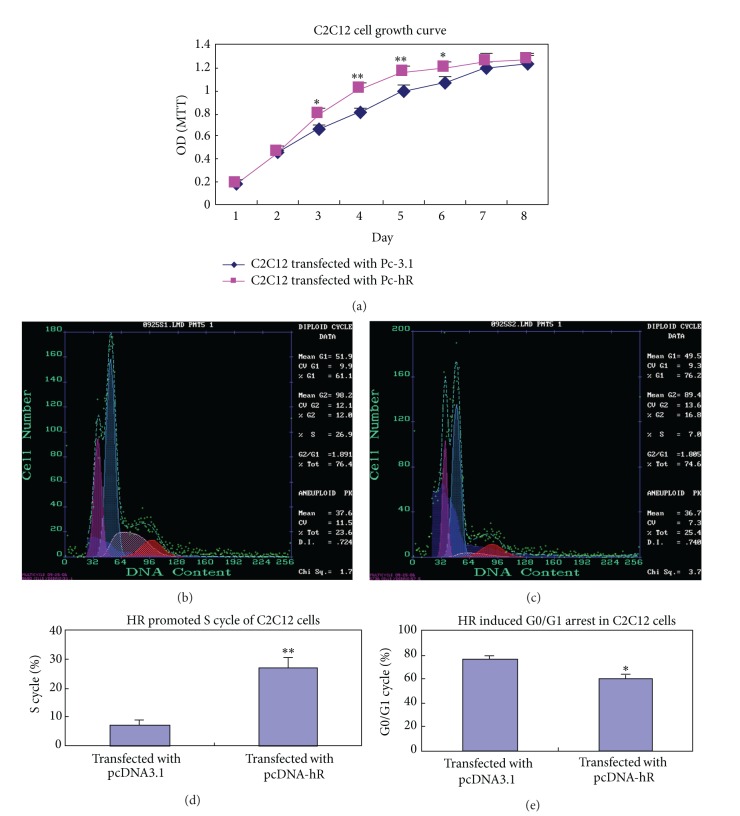
Effects of hR on proliferation and cell cycle of the C2C12 cells. (a) MTT showing significant increased absorbance of C2C12 cells transfected with Pc-hR compared with the control cells transfected with Pc-3.1. (b) Flow cytometry showing the cell cycle of C2C12 cells transfected with Pc-hR. (c) Flow cytometry showing cell cycle of C2C12 cells transfected with Pc-3.1. (d) Analysis of the percentages of S cycle. (e) Analysis of the percentages of G0/G1 cycle. Results are expressed as the means ± SEM of six measurements. *P < 0.05; **P < 0.01.

MTT assays were performed to determine cell proliferation. Compared with the control, proliferation of C2C12 cells transfected with Pc-hR was significantly promoted (Figure 2(a)). Overexpression of hR also significantly induced the S cycle accumulation in C2C12 cells. There were 26.9% of the S phase cells in C2C12 cells transfected with Pc-hR versus 7.0% in the control groups (*P* < 0.01) (Figures 2(b), 2(c), and 2(d)). At the same time, overexpression of hR significantly reduced the ratio of G0/G1 cycle in C2C12 cells transfected with Pc-hR (61.1% versus 76.2% in the control) (*P* < 0.01) (Figures 2(b), 2(c), and 2(e)).

### 3.3. Effects of hR on Myogenic Differentiation of C2C12 Cells

**Figure 3:**
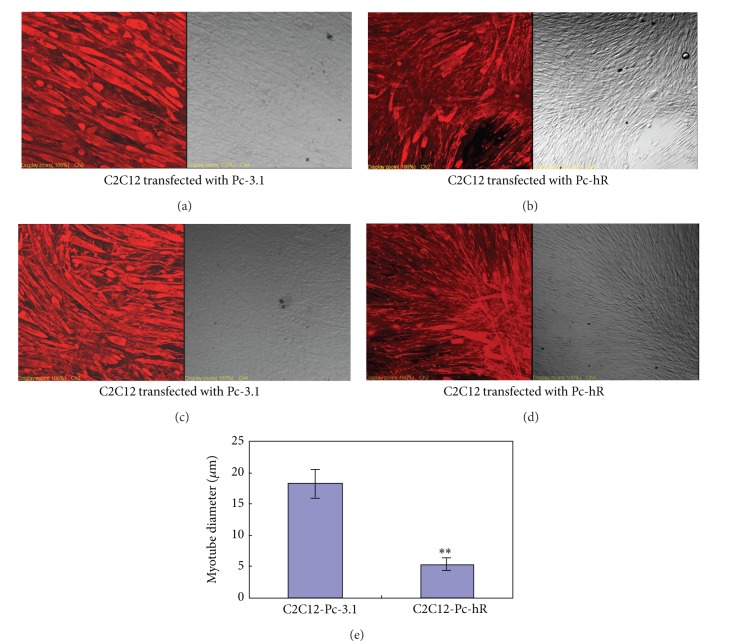
Effects of hR on myogenic differentiation of C2C12 cells (immunofluorescence staining). ((a) and (b)) The expression of desmin in C2C12 myotubes transfected with Pc-3.1 and transfected with Pc-hR vectors (magnification 200x). ((c) and (d)) The expression of myoglobin in C2C12 myotubes transfected with Pc-3.1 and transfected with Pc-hR vectors (magnification 200x). (e) Analysis of myotubes diameter. **P < 0.01.

Desmin is a muscle-specific intermediate filament protein which is expressed in both smooth and striated muscles. It and myoglobin are important markers of myogenic differentiation. Immunofluorescence staining was performed to detect the expression of desmin and myoglobin. Compared with the control cells transfected with Pc-3.1, expression of desmin and myoglobin decreased significantly in Pc-hR-transfected C2C12 myotubes (Figures 3(a), 3(b), 3(c), and 3(d)). HR also decreased the diameters of myotubes (*P* < 0.01) (Figure 3(e)). These data suggest that hR inhibited myogenic differentiation of C2C12 myoblasts.

### 3.4. Effects of hR on Glucose Uptake and Expression of Relevant Genes in C2C12 Myotubes

**Figure 4:**
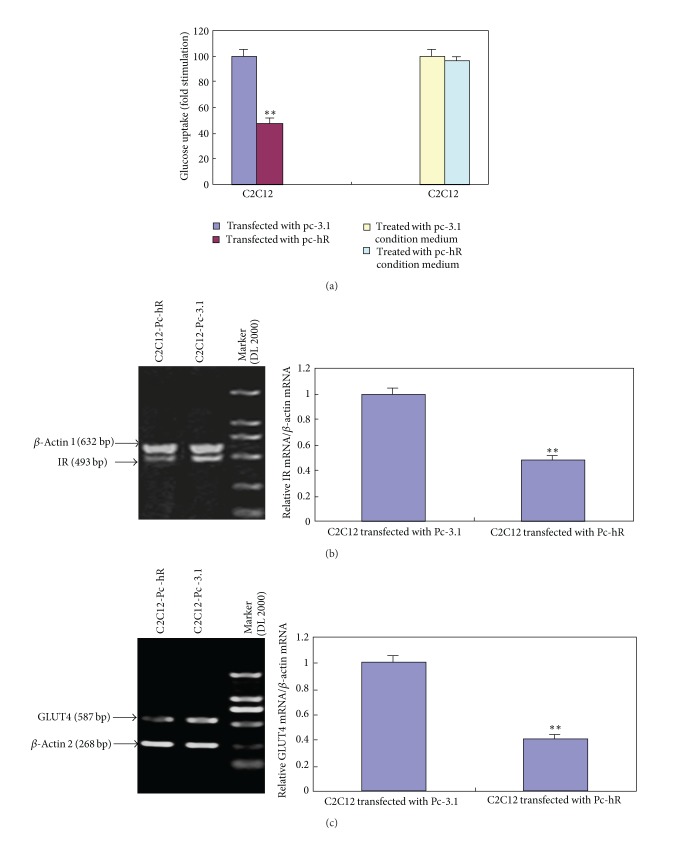
Effects of hR on glucose uptake and expression of relevant genes in C2C12 myotubes. (a) The effects of hR on glucose uptake in C2C12 myotubes. (b) Expression of insulin receptor (IR). (c) Expression of glucose transporter 4 (GLUT4). The amount of each target mRNA was normalized by the amount of *β*-actin mRNA and was expressed relative to the abundance of the target mRNA in cells transfected with Pc-3.1. **P < 0.01.

Data showed that insulin-stimulated glucose uptake in C2C12 myotubes transfected with Pc-hR was significantly decreased as compared with controls (Figure 4(a)). To further investigate the mechanisms, we examined the mRNA expression of IR and GLUT4 of C2C12 myotubes transfected with Pc-3.1 and Pc-hR plasmids. Semiquantitative RT-PCR analysis showed that expression of IR and GLUT4 mRNA decreased significantly in C2C12 myotubes transfected with Pc-hR as compared with the controls (*P* < 0.01) (Figures 4(b) and 4(c)).

To observe whether hR has a direct effect on insulin sensitivity of myocytes, condition mediums of COS7 cells transfected with Pc-3.1 and Pc-hR were used to culture C2C12 myotubes. The result showed that there was no significant difference in glucose uptake between the myotubes treated with Pc-hR condition medium and the myotubes treated with the Pc-3.1 condition medium (Figure 4(a)). 

## 4. Discussion

Since the discovery of resistin in 2001, there has been controversy on the role of resistin on glucose metabolism of skeletal muscles. It was reported not only that the resistin had no effect on insulin sensitivity of skeletal muscles [2, 20] but also that the resistin impaired insulin sensitivity of myocytes [3, 21, 22]. However, in the studies where resistin induced insulin resistance in myocytes, its mechanism was different or even contradictory. Fan et al. [3] and Palanivel et al. [21] thought that resistin impaired insulin sensitivity of rat skeletal muscle cells (differentiated from L6 myoblasts) by the inhibition of PI-3K insulin signal transduction pathway. Moon et al. [22] and his colleagues transfected recombined mouse resistin vector in L6 cells and the glucose uptake decreased significantly whereas PI-3K insulin signal transduction pathway was not affected. So they presumed that resistin degraded intrinsic activity of GLUTs. However, it was not mentioned whether resistin affects proliferation and differentiation of L6 myoblasts in these studies.

In the current study, we chose C2C12 myotubes as a cell culture model to test whether resistin can induce insulin resistance in muscle cells. C2C12 cells are immortalized cell lines from the C3H mouse skeletal muscle satellite cells, often used as the model of the study of the development and differentiation of muscle lineage [23], and insulin resistance is readily induced in this cell model with palmitate [24]. In order to observe the effect of resistin on muscle cell differentiation for a long time, resistin gene was expressed in C2C12 cells by the stable transfection technology so that it can offer the continuing effect of resistin on C2C12 cells. As well as to observe the direct effect of resistin on C2C12 myotubes, resistin gene was expressed in COS7 cells by the transient transfection technology so that it can offer the resistin protein direct effect on C2C12 myotubes.

That resistin inhibits adipogenic differentiation has been reported [5, 12, 14]. However, to our knowledge, its effect on myogenic differentiation has not been reported, but it was reported that Fizz1, which also belongs to Fizz family inhibited myogenesis [25]. Our data showed that hR dramatically stimulated the proliferation and increase of S phase cells whereas decreased G0/G1 phase cells. It also decreased diameter of myotubes and suppressed the expression of myogenic markers including desmin and myoglobin. These results indicate that resistin inhibits myogenesis and promotes proliferation of immature myocytes. Compared with the control cells, the glucose uptake and expression of IR and GLUT4 were dramatically decreased in C2C12 myotubes. However, compared with the controls, hR condition medium did not affect glucose uptake of C2C12 myotubes; nevertheless, it inhibits myogenesis and promotes proliferation of immature myocytes so that the population of mature myocytes and expression of IR and GLUT4 cutdown, which led to less glucose uptake. 

In a word, our results demonstrated for the first time that human resistin acted on cell cycle of myoblasts, inhibited myogenic differentiation, and promoted proliferation of myoblasts, which may be relevant to the glucose metabolic disorder.
